# Comprehensive Evaluation of Fruit Traits and Altitudinal Adaptability of 189 Wild *Camellia oleifera* Germplasms in East Guizhou, China

**DOI:** 10.3390/metabo16070512

**Published:** 2026-07-22

**Authors:** Tanming Ye, Bingqian Wu, Chengjiang Ruan

**Affiliations:** College of Environment and Resources, Dalian Minzu University, Dalian 116600, China; 18885669197@163.com (T.Y.); 19848106440@163.com (B.W.)

**Keywords:** *Camellia oleifera*, multi-trait comprehensive evaluation, principal component analysis (PCA), altitudinal adaptability, germplasm screening

## Abstract

**Highlights:**

**What are the main findings?**

- This study revealed rich variation in 21 traits among the 189 wild *Camellia oleifera* germplasms. The coefficients of variation (CV) were the highest for bioactive components, particularly beta + gamma-tocopherol (124.42%) and polyphenols (119.84%). In contrast, fatty acid composition showed lower variation, with total unsaturated fatty acids having the lowest CV (5.29%).
- Altitude had significant effects on specific traits. Germplasms at high altitude (800–1200 m) exhibited significantly higher seed oil content and kernel oil content than those at low altitude (400–800 m), with mean differences of 5.92 and 5.74 percentage points, respectively. However, altitude showed no significant effect on fruit morphological traits and most bioactive components (e.g., squalene and polyphenols).
- Superior germplasms were screened using a comprehensive evaluation function constructed via Principal Component Analysis (PCA). CL40 achieved the highest comprehensive score (Z*_n_* = 4.15), followed by MJX2 (Z*_n_* = 2.56). Notably, among the top 10 superior individuals, 8 were from the low-altitude group.

**What are the implications of the main findings?**

- The extreme phenotypic variation in key economic traits among wild *C. oleifera* germplasms in East Guizhou indicates that this region harbors valuable germplasm resources with high potential for breeding-oriented selection.
- Altitudinal variation drives differential responses in fruit traits of *C. oleifera*; therefore, this environmental constraint should be fully considered when evaluating germplasms across different altitudinal zones.
- The comprehensive evaluation method established in this study enables the quantitative ranking and precise screening of germplasms based on multi-trait data and may serve as a methodological reference for the germplasm evaluation of other woody oil crops in the same region.

**Abstract:**

**Background:** Eastern Tongren City, Guizhou Province, China, possesses abundant wild germplasm resources of *Camellia oleifera* Abel.; however, there is a lack of systematic evaluation, and the promotion of superior varieties is insufficient. This study aimed to evaluate 21 trait indices of 189 wild *C. oleifera* accessions from four regions in the Tongren area to clarify their variation characteristics, assess the effects of altitude on trait expression, and identify candidate germplasms with outstanding comprehensive performance. **Methods:** A total of 21 traits spanning fruit morphology, oil content, fatty acid composition, and bioactive components (tocopherols, squalene, and polyphenols) were measured. Principal Component Analysis (PCA) was employed to construct a comprehensive evaluation score (Z*_n_*) for quantitative ranking and screening. Trait differences between a low-altitude group (400–800 m, *n* = 139) and a high-altitude group (800–1200 m, *n* = 50) were compared using Welch’s *t*-test. **Results:** The germplasms exhibited abundant phenotypic variation, with coefficients of variation (CV) ranging from 5.29% (total unsaturated fatty acids) to 124.42% (beta + gamma-tocopherol). Bioactive components showed the highest variability, while fatty acid composition was relatively stable. Altitude had a significant effect on six of the 21 traits. Seed oil content and kernel oil content were significantly higher in the high-altitude group, with mean differences of 5.92 and 5.74 percentage points, respectively (both *p* < 0.001). However, oleic acid, total unsaturated fatty acids, fruit morphological traits, and most bioactive components showed no significant altitudinal differences (*p* > 0.05). The first five principal components explained 65.0% of the total variance. CL40 achieved the highest comprehensive score (Z*_n_* = 4.15), followed by MJX2 (Z*_n_* = 2.56). Among the top 10 individuals, eight were from the low-altitude group. **Conclusions:** This study revealed rich phenotypic variations and distinct altitudinal effects among wild *C. oleifera* germplasms in eastern Guizhou. The superior germplasms identified (such as CL40 and MJX2) can serve as candidate materials for locally adapted variety improvement. This study was primarily based on single-season phenotypic data, and the genetic stability of the selected germplasms should be validated through clonal trials and molecular marker analysis in future research.

## 1. Introduction

*Camellia oleifera* Abel. (Theaceae) is a perennial woody oil species endemic to China and ranks among the world’s four major woody oil plants alongside oil palm, olive, and coconut [1]. The oil extracted from its seeds contains approximately 90% unsaturated fatty acids, with oleic acid accounting for 74% to 87%, earning it the reputation of oriental olive oil [2]. Furthermore, the oil is rich in bioactive components such as squalene, tocopherols (vitamin E) and polyphenols, which confer antioxidant and anti-inflammatory properties and underpin its applications in premium edible oils, nutraceuticals, and cosmetics [3]. Tongren City in eastern Guizhou Province is one of the traditional suitable cultivation areas for *C. oleifera*, characterized by a long cultivation history, abundant wild germplasm resources, and a wide altitudinal range that creates diverse ecological niches for natural populations. However, these wild resources have long remained unevaluated and underutilized. The patterns of variation in fruit traits, oil quality, and functional components are largely unknown, and the existing resource advantage has yet to be translated into a tangible breeding advantage.

In recent years, studies on *C. oleifera* have advanced rapidly in genomics, breeding, and germplasm characterization [4], generating substantial baseline data on fruit phenotypic variation, seed oil content, and fatty acid composition. These studies have demonstrated that germplasms of *C. oleifera* exhibit considerable variations in key economic traits, including single fruit weight, pericarp thickness, seed yield rate, oil content, and fatty acid profile, thereby providing an important foundation for elite individual selection and varietal improvement. Evaluating the phenotypic diversity of germplasm resources constitutes the primary step in genetic improvement, because only by clarifying the extent and distribution of the trait variations within a population can target screening strategies be formulated. However, most existing studies have focused on single traits or a limited set of indicators and have been conducted predominantly in low-to-moderate altitude production areas, lacking both a systematic integration of phenotypic traits, oil quality, and functional components and an adequate consideration of the ecological effects associated with high-altitude environments. Altitude, as a critical ecological factor, has been shown to exert significant regulatory effects on the accumulation of secondary metabolites in plants. In several plant species, the environmental stresses associated with higher elevations, including increased ultraviolet radiation and lower temperatures, have been shown to promote the accumulation of phenolics, flavonoids, and other antioxidant metabolites [5]. In woody oil trees such as olive, altitude has also been shown to affect oil quality characteristics, including phenolic and tocopherol compositions [6]. Despite this body of evidence, a systematic multi-trait comprehensive evaluation of wild *C. oleifera* germplasms in eastern Guizhou has yet to be conducted. The mechanisms by which altitudinal gradients affect both economic traits and functional components of *C. oleifera* fruits remain poorly understood, and no screening system for elite germplasm across different altitudinal zones has been established. These gaps collectively impede the efficient exploration and utilization of wild *C. oleifera* germplasm resources in the region.

To address the aforementioned issues, this study collected 189 wild *C. oleifera* individuals from four counties in Tongren City, eastern Guizhou (Yuping County, Bijiang District, Songtao County, and Shiqian County). A total of 21 traits were systematically measured, encompassing fruit phenotypic traits, kernel oil content, fatty acid composition, and functional components (squalene, tocopherols, and polyphenols). PCA was employed to construct a comprehensive evaluation function for the quantitative ranking and screening of germplasms. In parallel, trait differences between two altitudinal groups (400–800 m vs. 800–1200 m) were compared to elucidate the effects of altitude on fruit traits. This study aimed to achieve three specific objectives: (1) to characterize the phenotypic variations in fruit traits, oil quality, and functional components among wild *C. oleifera* germplasms in eastern Guizhou; (2) to determine the direction and magnitude of altitudinal effects on key traits; and (3) to identify candidate germplasm with outstanding comprehensive performance. The findings are expected to provide a scientific basis for germplasm utilization and variety promotion in the eastern Guizhou oil tea industry.

## 2. Materials and Methods

### 2.1. Study Area and Sampling

**Figure 1:**
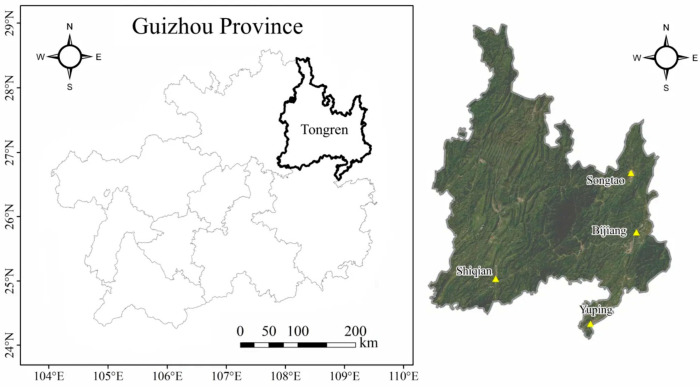
Geographic Distribution of *C. oleifera* Sampling Sites in Tongren, Guizhou Province.

The sampling sites for this study are located in Tongren City, eastern Guizhou Province, China, at 24.71° to 24.94° N and 105.79° to 106.05° E. A total of 189 wild *C. oleifera* individuals were collected from four counties or districts, namely Yuping County at altitudes of 400.6 to 542.3 m, Bijiang District at 477.1 to 589.1 m, Songtao County at 761.5 to 1172 m, and Shiqian County at 904.5 to 1162 m (Figure 1; Table 1). The terrain of this area belongs to typical low-hot valleys, and the climate is characterized by a subtropical warm humid monsoon climate. The average annual sunshine hours range from 1100 to 1400 h, the annual mean temperature ranges from 16.4 to 16.9 °C with an extreme minimum of approximately −4 °C, the average frost-free period is about 345 days, and the annual precipitation ranges from 1096 to 1440 mm. The soil at the sampling sites is slightly acidic. Based on the altitudinal distribution, the individuals were classified into a low-altitude group (400 to 800 m, *n* = 139) and a high-altitude group (800 to 1200 m, *n* = 50). Mature fruits were harvested in mid-October each year, with thirty fruits randomly collected from each sample tree and shipped to the laboratory for experimental analysis (Table S2).

### 2.2. Trait Measurement

#### 2.2.1. Fruit Phenotypic Traits

The phenotypic traits of *C. oleifera* fruits were evaluated following a previously described protocol [7]. For each individual, fruits were thoroughly mixed, and five fruits were randomly selected for analysis, and all measurements were performed in triplicate. The fruit length, fruit diameter, and pericarp thickness were measured using a digital electronic caliper (±0.01 mm). The fresh fruit weight and dry seed weight were recorded using an electronic balance (±0.001 g). The maximum single fruit weight was recorded as the heaviest fruit among the sampled fruits. The following derived indices were calculated:

$$Fruit\;shape\;index\;=\;\frac{\;fruit\;length}{fruit\;diameter}$$

$$Seed\;yield\;rate\;of\;fresh\;fruit\;(\%)=\frac{fresh\;seed\;weight}{fruit\;weight}\times 100\%$$

$$Seed\;yield\;rate\;of\;dried\;fresh\;fruit\;(\%)=\frac{dry\;seed\;weight}{fresh\;seed\;weight}\times 100\%$$

#### 2.2.2. Seed Oil Content and Kernel Oil Content

The seed oil content was determined using a low-field nuclear magnetic resonance analyzer, and the average of three measurements was taken [8]. The oil content was determined using the Soxhlet extraction method [9]. Seeds were dehulled to obtain kernels, which were dried to a constant weight at 60 °C. The dried kernels were ground into a homogeneous powder using a high-efficiency grinder. Approximately 20 g of powder (±0.0001 g) was wrapped in a pre-extracted and pre-dried filter paper thimble and placed into a Soxhlet extractor. Petroleum ether (30–60 °C boiling range) was used as the extraction solvent at 50 °C under reflux for 6–8 h until the extract became nearly colorless. The solvent was removed by rotary evaporation at 55 °C. The residual oil was quantitatively transferred to a pre-dried and tared glass weighing dish, dried at 60 °C to constant weight, and weighed. The oil content was calculated as follows:

$$Oil\;content\;(\%)=\left(\frac{m_{oil}}{m_{sample}}\right)\times 100\%$$

where $m_{oil}$ is the mass of extracted oil (g) and $m_{sample}$ is the mass of kernel powder (g).

#### 2.2.3. Fatty Acid Composition

A total of seven fatty acid parameters were determined in this study, including palmitic acid, stearic acid, oleic acid, linoleic acid, linolenic acid, gadoleic acid, and total unsaturated fatty acids.

The fatty acid composition was determined by GC-MS following base- and acid-catalyzed transesterification [10]. Briefly, 200 μL of oil was dissolved in 1 mL of n-hexane and mixed with 500 μL of 1 mol/L KOH methanol. The mixture was incubated at 60 °C for 30 min. After cooling, 1 mL of BF3 methanol (10–14% *w*/*v*) was added and heated at 60 °C for 30 min. Following esterification, 1 mL of saturated NaCl and 2 mL of n-hexane were added. The mixture was vortexed vigorously to facilitate phase separation, and the upper organic layer was collected and filtered through a 0.22 μm organic-phase syringe filter. The percentage content of each fatty acid was calculated by peak area normalization.

GC-MS conditions: DB-23 column (60 m × 0.25 mm, 0.25 μm); carrier gas, helium at 1 mL/min; injector, 230 °C split ratio, 20:1; oven program, 50 °C (1 min) → 15 °C/min → 200 °C (28 min) → 10 °C/min → 220 °C (3 min); EI mode at 230 °C; transfer line, 215 °C; *m*/*z*, 45–400.

#### 2.2.4. Tocopherol Content

Lipid-soluble antioxidants including tocopherols and tocotrienols in *C. oleifera* oil were determined by RP-HPLC-FLD [11]. Briefly, 0.5 g of oil was weighed into a 50 mL screw-cap centrifuge tube and mixed with 0.25 g of ascorbic acid, 0.025 g of BHT, 7.5 mL absolute ethanol, and 2.5 mL of 1:1 (*v*/*v*) aqueous KOH. The mixture was saponified in a thermostatic shaker (BOXUN, Shanghai, China) at 45 °C for 60 min. After cooling, 7.5 mL of ultrapure water was added, and the mixture was extracted with 12.5 mL of petroleum ether (30–60 °C) by vortex mixing for 5 min. The upper organic layer was collected, and the aqueous phase was re-extracted with an additional 12.5 mL of petroleum ether. The combined organic phase was washed twice with 13 mL of ultrapure water. The final extract was transferred to a glass test tube and gently evaporated to near dryness under nitrogen at 40 °C. The residue was reconstituted in 1.0 mL of HPLC-grade methanol, vortexed thoroughly, filtered through a 0.22 μm organic-phase syringe filter, and stored at 4 °C in the dark for analysis within 24 h.

HPLC conditions: C18 column (150 mm × 4.6 mm, 5 μm); methanol isocratic; 0.8 mL/min; column temperature, 20 °C; Ex 294 nm/Em 328 nm; injection volume, 1 μL. α-, β + γ-, and δ-tocopherol were identified by retention time comparison with authentic standards. Calibration curves were constructed by plotting peak areas against concentrations of mixed standard solutions, and the linearity was evaluated by R2. The tocopherol content was expressed as milligrams per kilogram of oil (mg/kg).

#### 2.2.5. Squalene Content

The squalene in *C. oleifera* oil was determined by GC-MS following saponification and liquid extraction [12]. Briefly, 0.5 g of oil was weighed into a screw-cap glass tube (PADAMAN, Nantong, China) and mixed with 25 mg of ascorbic acid, 2.5 mg of BHT, 7.5 mL of ethanol, and 2.5 mL of a 1:1 (*v*/*v*) aqueous KOH solution. The mixture was saponified at 45 °C for 60 min with intermittent vortexing. After cooling, the squalene was extracted with petroleum ether (30–60 °C), the aqueous layer was re-extracted once, and the combined organic phase was washed twice with ultrapure water. The final extract was transferred to a glass test tube and gently evaporated to near dryness under nitrogen at 40 °C. The residue was reconstituted in 1 mL of HPLC-grade n-hexane, vortexed thoroughly, and filtered through a 0.22 μm organic-phase syringe filter (JINTENG, Tianjin, China). The GC-MS conditions were identical to those described in Section 2.2.3. Squalene was identified by comparing its retention time and mass spectrum with an authentic standard (≥99% purity). Calibration curves were constructed, and the squalene content is expressed as mg/kg oil. All analyses were performed in triplicate.

#### 2.2.6. Total Polyphenol Content

The total polyphenol content (TPC) in *C. oleifera* seed oil was determined using the Folin–Ciocalteu (Solarbio, Beijing, China) method [13]. Briefly, 2 g of oil in 6 mL of n-hexane was loaded onto a diol-SPE cartridge preconditioned with 5 mL of methanol and 5 mL of n-hexane at a flow rate of approximately 1 mL/min. After washing with 10 mL of n-hexane, polyphenols were eluted with 10 mL of HPLC-grade methanol. The eluate was evaporated under nitrogen at 45 °C, reconstituted in 2 mL of methanol–water (50:50, *v*/*v*), vortexed for 1 min, and stored at −18 °C for 16 h. The mixture was centrifuged (1000× *g*, 5 min, 4 °C) and the supernatant collected. For colorimetric analysis, 1 mL of extract was mixed with 0.5 mL of Folin–Ciocalteu reagent, 2.0 mL of 7.5% Na_2_CO_3_, and 6.5 mL of ultrapure water, vortexed for 1 min, incubated at 70 °C for 30 min in the dark, and cooled to room temperature. The absorbance was measured at 760 nm using a 10 mm quartz cuvette. The TPC is expressed as mg gallic acid equivalents per kg oil (mg GAE/kg), based on a gallic acid calibration curve (0–500 mg/L, R^2^ > 0.999). All chemical analyses were performed in triplicate and are reported as the mean ± SD. Method validation parameters for all assays are summarized in Supplementary Table S3.

### 2.3. Statistical Analysis

All statistical analyses were performed in R (version 4.6.1). Sampling maps were generated using ArcMap 10.8 (ESRI, Redlands, CA, USA) Twenty-one traits were measured across four categories: fruit phenotypic traits, kernel oil content, fatty acid composition, and bioactive components (tocopherols, squalene, and polyphenols).

Zero values representing unmeasured traits (identified when an entire trait group was simultaneously zero, e.g., all three fruit morphological indices or both oil content measures) were recoded as missing (NA), while sporadic zero values in δ-tocopherol, squalene, and polyphenols, likely reflecting concentrations below the detection limit, were retained.

Descriptive statistics, correlation analysis, and altitudinal comparisons were performed using available observations for each trait without imputation. PCA, however, requires a complete data matrix across all traits for each individual. Of the 189 samples, 14 lacked the entire suite of oil and fatty acid measurements, with a missing rate exceeding 57%, and were therefore excluded. Among the remaining 175 samples, 54 had partial missing values, mainly in three fruit morphological traits, with a per-sample missing rate of approximately 14%. These missing values were imputed using predictive mean matching, referred to as PMM, via the mice package [14], with five imputation sets generated over 20 iterations, yielding a complete 175 × 21 data matrix for PCA.

The PCA was validated by the KMO test and Bartlett’s sphericity test, confirming its applicability for subsequent analysis. The communalities of most traits exceeded 0.5, indicating adequate representation by the five retained PCs, although seed yield rate of fresh fruit (0.25) and polyphenol content (0.28) were less well captured.

Descriptive statistics (mean, SD, range, and CV) were calculated for each trait using valid observations. Pearson correlation coefficients were computed using pairwise complete observations, with significance assessed at α = 0.05, 0.01, and 0.001. Differences between the two altitudinal groups were evaluated using Welch’s *t*-test.

Hierarchical cluster analysis was performed on the 21 traits using the Pearson correlation distance (1 − *r*) and UPGMA linkage. The optimal number of clusters (*K* = 5) was determined by evaluating the biological interpretability and silhouette coefficients across *K* = 3–8. The first five PCs, collectively explaining approximately 65% of the total variance, were retained based on the cumulative variance explained and biological interpretability. A comprehensive evaluation score (Z*_n_*) was calculated following the PCA-based method established for *C. oleifera* germplasm selection [15]:

$$Z_{n}\;=\;\frac{R_{1}{PV}_{1}\;+\;R_{2}{PV}_{2}\;+\;R_{3}{PV}_{3}\;+\;R_{4}{PV}_{4}\;+\;R_{5}{PV}_{5}}{C}$$

where *PV*_1_–*PV*_5_ are individual PC scores, *R*_1_–*R*_5_ the corresponding variance contribution rates, and *C* the cumulative contribution rate of the five retained PCs. Individuals were ranked by Z*_n_* in descending order to identify candidate germplasms with superior comprehensive performance.

All figures were generated using the ggplot2, ggtree, aplot, and ggrepel packages in R.

## 3. Results

### 3.1. Phenotypic Variation Among Germplasms

The descriptive statistics for the 21 traits measured across 189 wild *C. oleifera* germplasms are presented in Table 2. The coefficient of variation (CV) ranged from 5.29% (total unsaturated fatty acids) to 124.42% (beta + gamma-tocopherol). The traits with the highest CVs were beta + gamma-tocopherol (124.42%), polyphenol (119.84%), maximum single fruit weight (97.29%), squalene (96.67%), and delta-tocopherol (72.61%). The traits with the lowest CVs were total unsaturated fatty acids (5.29%), oleic acid (6.30%), and fruit shape index (9.91%).

Oleic acid was the predominant fatty acid, with a mean content of 68.45% (range of 55.26–82.53%), followed by palmitic acid (15.55%) and linoleic acid (7.72%). The kernel oil content averaged 39.67% (range of 14.74–64.12%), and seed oil content averaged 24.83% (range, 6.58–41.20%). Among the bioactive components, alpha-tocopherol was the most abundant vitamin E form (mean, 40.19 mg/kg), squalene averaged 90.12 mg/kg (range of 0–584.47 mg/kg), and the polyphenol content averaged 21.67 mg GAE/kg.

### 3.2. Correlation Analysis

**Figure 2:**
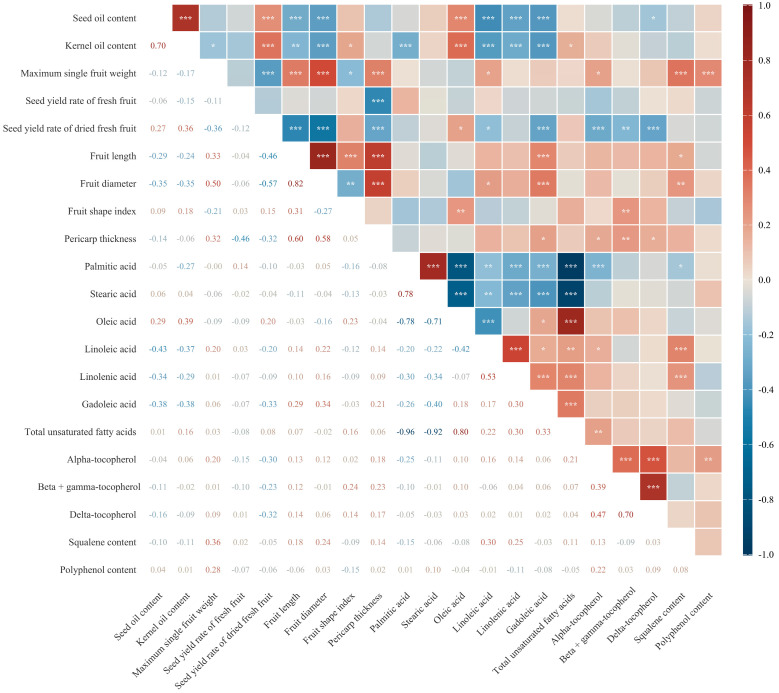
Heatmap of Pearson Correlation Matrix for 21 Fruit Traits in *C. oleifera*. Significance levels are indicated as: *, *p* < 0.05; **, *p* < 0.001, ***, *p* < 0.0001.

Pearson correlation analysis revealed complex interrelationships among the 21 traits (Figure 2). The strongest positive correlations were observed between fruit length and fruit diameter (*r* = 0.82, *p* < 0.001), palmitic acid and stearic acid (*r* = 0.78, *p* < 0.001), seed oil content and kernel oil content (*r* = 0.70, *p* < 0.001), and beta + gamma-tocopherol and delta-tocopherol (*r* = 0.70, *p* < 0.001). The strongest negative correlations were found between palmitic acid and total unsaturated fatty acids (*r* = −0.96, *p* < 0.001) and between stearic acid and total unsaturated fatty acids (*r* = −0.92, *p* < 0.001).

Oleic acid showed a strong positive correlation with total unsaturated fatty acids (*r* = 0.80, *p* < 0.001) and strong negative correlations with palmitic acid (*r* = −0.78, *p* < 0.001) and stearic acid (*r* = −0.71, *p* < 0.001). Oil content traits generally showed no significant correlation with tocopherol content (*p* > 0.05), except for a weak negative correlation between seed oil content and delta-tocopherol (*r* = −0.16, *p* = 0.030). The squalene and polyphenol content were not significantly correlated with fatty acid composition or oil content traits.

### 3.3. Altitudinal Effects on Trait Variation

**Figure 3:**
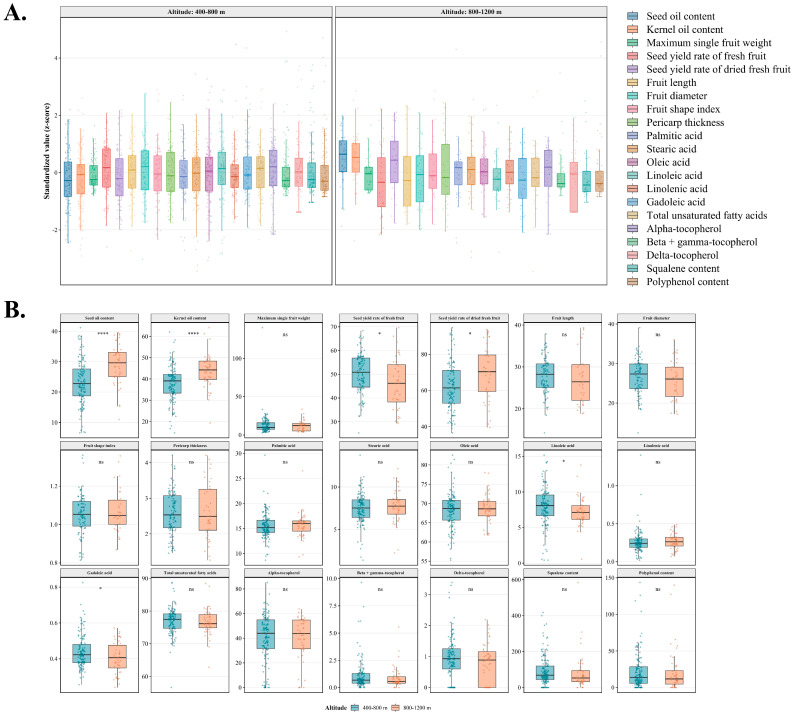
Comparison of 21 fruit traits between low-altitude (400–800 m) and high-altitude (800–1200 m) *C. oleifera* germplasm groups. (**A**) Distribution of standardized trait values (z-scores) across the two altitude groups. (**B**) Pairwise comparisons of individual traits between altitude groups using Welch’s *t*-test. Significance levels are indicated as: ns, not significant; *, *p* < 0.05; ****, *p* < 0.0001.

Welch’s *t*-test showed that 6 of the 21 traits differed significantly between the low-altitude (400–800 m) and high-altitude (800–1200 m) groups (Figure 3A,B). The seed oil content (*p* < 0.001) and kernel oil content (*p* < 0.001) were significantly higher in the high-altitude group (29.09% and 43.80%) than in the low-altitude group (23.17% and 38.07%), with mean differences of 5.92 and 5.74 percentage points, respectively. The dry seed yield rate was significantly higher at high altitude (69.47% vs. 62.43%, *p* = 0.010), whereas the fresh seed yield rate was significantly lower (45.98% vs. 50.37%, *p* = 0.023). Linoleic acid (*p* = 0.044) and gadoleic acid (*p* = 0.032) showed significant decreases at higher altitude.

The remaining 15 traits, including fruit morphological traits (fruit length, fruit diameter, fruit shape index, pericarp thickness, maximum single fruit weight), oleic acid, total unsaturated fatty acids, all three tocopherols, squalene, and polyphenol, showed no significant altitudinal differences (*p* > 0.05).

### 3.4. Hierarchical Cluster Analysis

**Figure 4:**
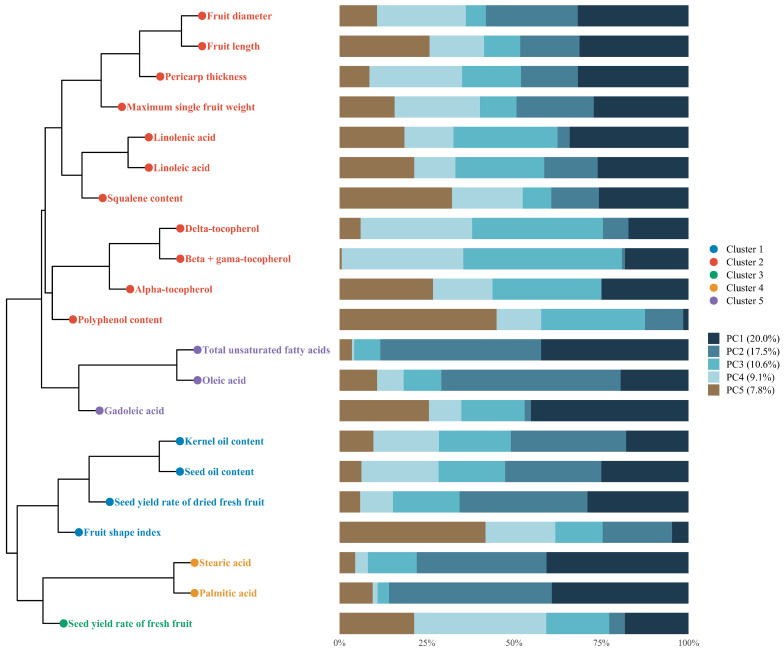
Hierarchical Clustering Analysis of 21 Traits Based on Similarity.

Hierarchical cluster analysis based on Pearson correlation distance grouped the 21 traits into five clusters (Figure 4). Cluster 1 comprised seed oil content, kernel oil content, dry seed yield rate, and fruit shape index. Cluster 2, the largest cluster, included maximum single fruit weight, fruit length, fruit diameter, pericarp thickness, linoleic acid, linolenic acid, alpha-tocopherol, beta + gamma-tocopherol, delta-tocopherol, squalene, and polyphenols. Cluster 3 contained only fresh seed yield rate. Cluster 4 grouped palmitic acid and stearic acid. Cluster 5 consisted of oleic acid, gadoleic acid, and total unsaturated fatty acids.

The PCA loading profiles displayed alongside the dendrogram (Figure 4) showed that traits within the same cluster exhibited similar loading patterns across the five retained PCs, while traits in different clusters showed distinct loading signatures. Clusters 4 and 5 each contained traits from a single biochemical category, whereas Cluster 2 contained traits spanning fruit morphology, polyunsaturated fatty acids, tocopherols, and other bioactive components.

### 3.5. Principal Component Analysis and Comprehensive Evaluation

PCA was performed on 175 individuals (14 samples were excluded due to missing oil-related data; missing values were imputed using mice PMM). Seven PCs had eigenvalues greater than 1; the first five were retained based on interpretability and cumulative variance, collectively explaining 65.0% of the total variance (Table 3). PC1 (20.0%) was primarily loaded on fruit morphological traits (fruit length, 0.60; fruit diameter, 0.58; pericarp thickness, 0.50) and fatty acid traits (total unsaturated fatty acids, 0.66; stearic acid, −0.66; palmitic acid, −0.61). PC2 (17.5%) had strong loadings on oleic acid (−0.85), palmitic acid (0.73), kernel oil content (−0.60), and dry seed yield rate (−0.50). PC3 (10.6%) was loaded on beta + gamma-tocopherol (0.65), delta-tocopherol (0.63), and alpha-tocopherol (0.49). PC4 (9.1%) had high loadings on fruit diameter (0.47) and pericarp thickness (0.42), and negative loadings on delta-tocopherol (−0.53) and beta + gamma-tocopherol (−0.49). PC5 (7.8%) was loaded on fruit shape index (−0.50) and fruit length (−0.49), with positive loadings on squalene (0.43), alpha-tocopherol (0.42), and polyphenol (0.42).

**Figure 5:**
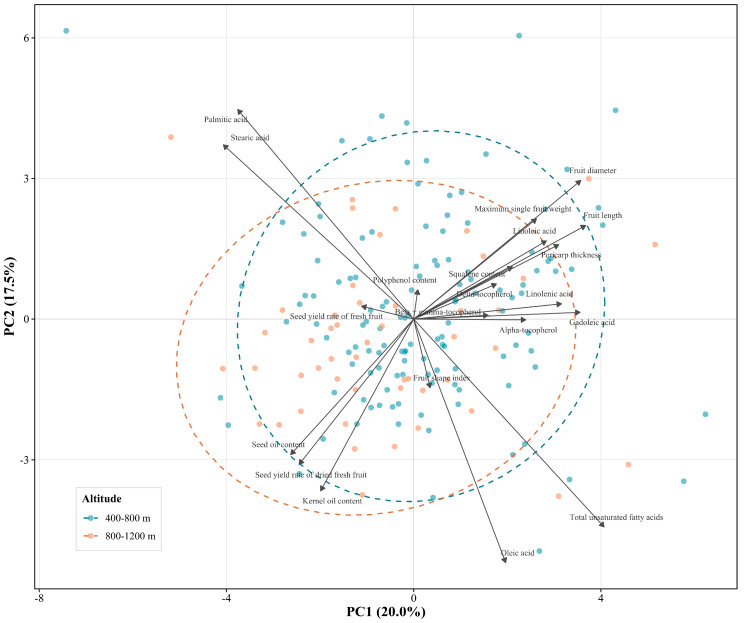
Biplot of the 95% confidence ellipses for two altitudinal groups on the PC1-PC2 plane.

The PCA biplot (Figure 5) showed overlap between the 95% confidence ellipses of the two altitudinal groups on the PC1-PC2 plane.

**Figure 6:**
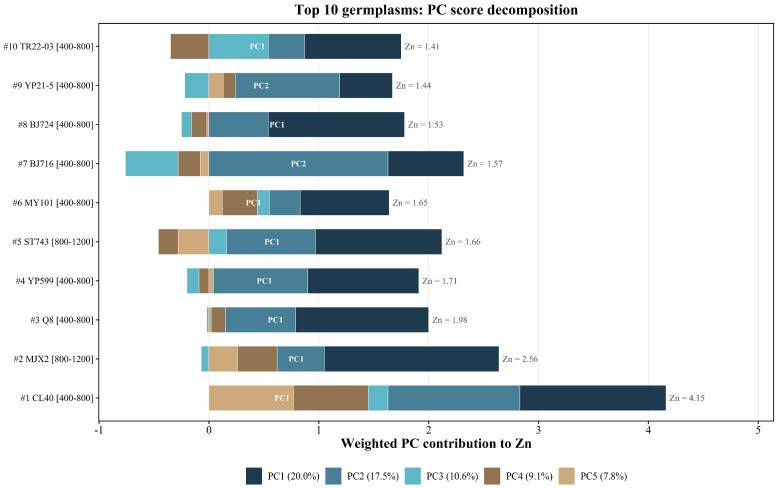
Score decomposition of the top 10.

The comprehensive evaluation scores (Z*_n_*) ranged from −1.93 to 4.15. The top-ranked individual was CL40 (Z*_n_* = 4.15) from the low-altitude group, followed by MJX2 (Z*_n_* = 2.56) from the high-altitude group and Q8 (Z*_n_* = 1.98) from the low-altitude group. Among the top 10 individuals, 8 were from the low-altitude group and 2 from the high-altitude group; among the top 20, 15 were from the low-altitude group and 5 from the high-altitude group. PC score decomposition of the top 10 individuals (Figure 6) revealed that PC1 (fruit morphology and fatty acid composition) was the dominant contributor to Z*_n_* for 8 of the 10 top-ranked germplasms, while PC2 (oil content and oleic acid) was the primary driver for BJ716 and YP21-5. Notably, CL40 exhibited balanced contributions across multiple PCs (PC1 = 1.33, PC2 = 1.20, PC4 = 0.68, PC5 = 0.77), whereas most other top-ranked individuals were predominantly driven by a single component. The complete ranking is provided in Supplementary Table S1.

## 4. Discussion

The *C. oleifera* industry has become a strategic sector for ensuring national grain and oil security and promoting rural revitalization in mountainous regions of southern China [16]. However, in key production areas such as Tongren, Guizhou, improved varieties introduced from other provinces frequently fail to establish or to achieve expected productivity under local conditions. Yang et al. [7] noted that making full use of local germplasm resources to screen and cultivate superior cultivars with strong adaptability is the most economical and effective breeding approach. This study represents the first systematic phenotypic characterization and comprehensive evaluation of 189 wild *C. oleifera* germplasms from the Tongren region, covering 21 traits spanning fruit morphology, oil content, fatty acid composition, and bioactive components.

### 4.1. Phenotypic Diversity and Trait Variation

The 189 wild germplasms exhibited substantial phenotypic diversity, with CVs ranging from 5.29% for total unsaturated fatty acids to 124.42% for beta + gamma-tocopherol (Table 2). Bioactive and morphological traits such as beta + gamma-tocopherol (124.42%), polyphenols (119.84%), maximum single fruit weight (97.29%), and squalene (96.67%) showed far greater variation than fatty acid composition traits, indicating that these components harbor substantial phenotypic variation available for selection. These bioactive components are increasingly recognized for their antioxidant, anti-inflammatory, and anticancer properties [17] and their extensive variability presents opportunities not only for edible oil production but also for the development of high-value cosmetic and pharmaceutical products [18]. The consistently low CV of oleic acid (6.30%) aligns with similarly low values reported in other populations [19], indicating that oleic acid content is relatively stable across *C. oleifera* germplasms. However, the mean oleic acid content in the present wild population (68.45%) was notably lower than that reported in other *C. oleifera* populations [18,19,20], underscoring both the potential for improvement through selection and the broader genetic base available in the Tongren wild population.

The kernel oil content averaged 39.67% (range, 14.74–64.12%), comparable to the range observed in Wangmo red ball *C. oleifera* from Guizhou’s low-heat valley [21]. Squalene averaged 90.12 mg/kg (range 0–584.47 mg/kg), and polyphenol content averaged 21.67 mg GAE/kg, both showing extensive variation as reflected in their high CVs.

### 4.2. Trait Correlations and Their Biological Basis

The correlation analysis (Figure 2) and hierarchical clustering (Figure 4) revealed trait groupings that reflect underlying biosynthetic pathways. The strong negative correlations between oleic acid and both palmitic acid (*r* = −0.78) and stearic acid (*r* = −0.71), and the inverse relationship between palmitic acid and total unsaturated fatty acids (*r* = −0.96), can be explained by the fatty acid desaturation pathway, in which *SAD* and *FAD2* sequentially regulate the conversion from saturated to unsaturated fatty acids [22]. Previous transcriptomic studies in *C. oleifera* have shown that the coordinated regulation of these enzymes during seed development is critical for oleic acid accumulation [23], and the phenotypic correlations observed in the present study are in line with that mechanism. The clustering of saturated fatty acids (Cluster 4) separately from unsaturated fatty acids (Cluster 5) aligns with this metabolic framework. The independence of oil content traits from tocopherol content (*p* > 0.05), with only a weak negative correlation between seed oil content and delta-tocopherol, indicates that the simultaneous improvement of oil yield and vitamin E content through breeding is feasible without encountering trait antagonism.

### 4.3. Altitudinal Effects on Trait Variation and Ecological Implications

Among the 21 traits examined, only six showed significant differences between the two altitude groups (Figure 3A,B), indicating that most traits are not significantly affected by the altitudinal gradient examined in this study. The most notable altitudinal effect was the significantly higher seed oil content and kernel oil content in the high-altitude group. In *C. chekiangoleosa*, oil accumulation in mature seeds was found to occur at the cost of consuming soluble sugars, starch, and proteins, and endocarp tissue development also varied with altitude during fruit ripening [24]. Whether the cooler temperatures at higher altitudes directly drive this metabolic shift in *C. oleifera* remains to be investigated. The higher dry seed yield rate accompanied by a lower fresh seed yield rate observed at high altitude may reflect greater endocarp development at higher elevation, which serves a protective function for seed kernel development under low-temperature conditions.

Although oil content increased with altitude, the oleic acid content and total unsaturated fatty acid content remained stable across the two altitude groups (Figure 3B). This finding is noteworthy given that Zeng and Endo [25] demonstrated significant differences in fatty acid composition among *C. oleifera* from different cultivars and planting regions across China. The stability of oleic acid across the altitudinal gradient in the present study suggests that, compared to cultivar identity and broader geographic origin, altitude alone has limited influence on this particular fatty acid in the Tongren population. The significant decrease in linoleic acid at higher altitude, coupled with no change in oleic acid, suggests that individual fatty acid species may differ in their sensitivity to environmental variation. It is well established that growing region, climate, and cultivar type can have tremendous impacts on the properties of camellia seed oil [26,27] and the present study adds to this body of knowledge by demonstrating that, within a single production region, the effect of altitude on oil quantity is substantial, while the major oil quality indicators, particularly oleic acid and total unsaturated fatty acid content, remain unaffected.

### 4.4. Comprehensive Evaluation and Breeding Implications

PCA is an effective method for reducing the dimensionality of large datasets while maximizing interpretability [28]. In this study, the first five principal components explained 65.0% of the total variance (Table 3). The PCA biplot (Figure 5) showed overlap between the 95% confidence ellipses of the two altitudinal groups on the PC1-PC2 plane, and the comprehensive evaluation identified CL40, MJX2, and Q8 as the three highest-ranking germplasms. Similar multivariate approaches have been widely applied across *C. oleifera* producing regions, with studies employing M-TOPSIS based on PCA in Sichuan [29] and PCA-based evaluation in northwestern Guizhou [15] each identifying a small set of top-performing individuals from large wild populations.

The PC score decomposition analysis (Figure 6) adds an important layer of interpretation by revealing the specific trait dimensions driving each individual’s ranking. The finding that PC1 (fruit morphology and fatty acid composition) was the dominant contributor for 8 of the 10 top-ranked germplasms, while PC2 (oil content and oleic acid quality) primarily drove the scores of BJ716 and YP21-5, revealing meaningful heterogeneity among the top selections. This heterogeneity has direct implications for germplasm utilization, because it allows researchers to identify individuals with distinct trait advantages suited to different production objectives. Eight of the top 10 individuals originated from the low-altitude group, with two individuals from the high-altitude group among them. Given that the high-altitude group showed significantly elevated oil content, MJX2 is of particular interest as a promising candidate that combines a high comprehensive score with the oil content advantage characteristic of high-altitude populations.

### 4.5. Limitations, Management Recommendations, and Future Perspectives

Several limitations should be acknowledged. All phenotypic data were collected in a single growing season, making it impossible to partition the observed variation into genetic and environmental components. Multi-year evaluations, such as the four-year scheme adopted by Zheng et al. [30] for 302 accessions in Ya’an, would strengthen the reliability of the rankings. The relatively high proportion of missing morphological data (up to 35%) necessitated the use of multiple imputations, which introduces additional uncertainty that may affect individual rankings.

The results have direct implications for germplasm management in the Tongren region. Wan et al. [15] highlighted the shortage of varieties suitable for high-altitude regions as an urgent problem. The finding that oil content was significantly higher at high altitude while the major oil quality indicators remained unaffected suggests that high-altitude sites in Tongren may be particularly suitable for oil-oriented production, though multi-year data are needed to confirm this pattern. Given the repeated failure of externally introduced cultivars to adapt to local conditions, a locally driven breeding strategy that exploits the extensive variation documented here may prove more effective than continued reliance on cultivars developed elsewhere. We recommend that the top-ranked germplasms be included in clonal archives and field performance trials before large-scale promotion. Kong et al. [31] have provided a framework for the survey, collection, and conservation of wild *C. oleifera* in southwest China, and their approach could serve as a model for establishing a dedicated conservation program for the Tongren population.

Future research should incorporate molecular marker analysis to confirm the genetic distinctness of top-ranked germplasms and quantify population structure across the altitudinal gradient. Zhu et al. [32] demonstrated the value of combining fruit phenotype and SSR data for constructing a core germplasm collection of *C. oleifera* in Guizhou Province, and a similar approach would facilitate more efficient conservation. The altitudinal effect on linoleic acid observed here, together with previous reports that *FAD2* expression during camellia seed development closely tracks linoleic acid accumulation [33], suggests that transcriptomic studies across the altitudinal gradient would help elucidate the molecular basis of the observed phenotypic patterns. Multi-criteria decision-making methods such as the Analytic Hierarchy Process combined with entropy weighting could also be explored to incorporate breeding-objective-specific priorities into the evaluation framework.

## 5. Conclusions

This study systematically evaluated the phenotypic traits of 189 wild *C. oleifera* germplasms in eastern Guizhou, revealing their rich variation characteristics and altitudinal effects. The main conclusions are as follows. The germplasms exhibited abundant trait variation, with bioactive components (beta + gamma-tocopherol and polyphenols) showing the highest coefficients of variation. Altitude had significant effects on certain traits. Germplasms at high altitude (800–1200 m) had significantly higher seed oil content and kernel oil content than those at low altitude (400–800 m). Superior germplasms, such as CL40 and MJX2, were screened through comprehensive evaluation. They can serve as candidate materials for improving *C. oleifera* varieties, providing high-quality breeding resources for the Tongren region.

This study was primarily based on phenotypic data from individual wild plants, and their superior traits might be influenced by specific micro-environments, with their genetic stability yet to be verified. Future research should validate the genetic stability of the selected superior germplasms under controlled environmental conditions (e.g., by establishing stool beds or conducting clonal tests) and combine molecular marker techniques to further elucidate the genetic basis of their superior traits.

## Figures and Tables

**Table 1:** Information of Sampling Sites for Oil-tea Camellia Germplasm Resources in Tongren City, Guizhou Province.

Sample Name	Germplasm Source	Germplasm Type	Altitude (m)	Annual Precipitation (mm)	Annual Avg. Temp. (°C)
YP504–YP570	Yuping County	Wild seedling	400.6–542.3	1100–1200	16.4
BJ716–BJ741	Bijiang District	Wild seedling	477.1–589.1	1250–1440	16.9
ST743–ST759	Songtao County	Wild seedling	761.5–1172	1300–1400	16.5
SQ760–SQ794	Shiqian County	Wild seedling	904.5–1162	1096–1200	16.8

**Table 2:** Descriptive Statistics of Traits.

Trait	N	Min	Max	Median	SD	CV (%)
Seed oil content	175	6.58	41.20	25.03	7.43	29.94
Kernel oil content	175	14.74	64.12	40.58	8.57	21.59
Maximum single fruit weight	147	2.78	140.31	10.04	12.46	97.29
Seed yield rate of fresh fruit	153	25.37	69.64	49.19	9.18	18.65
Seed yield rate of dried fresh fruit	153	36.54	94.78	63.78	13.96	21.71
Fruit length	128	14.30	39.38	27.87	4.95	17.82
Fruit diameter	128	12.50	39.08	26.53	4.60	17.42
Fruit shape index	128	0.81	1.36	1.05	0.10	9.91
Pericarp thickness	153	1.26	4.21	2.53	0.66	25.20
Palmitic acid	175	8.67	29.65	15.44	2.55	16.37
Stearic acid	175	1.40	13.74	7.63	1.79	23.72
Oleic acid	175	55.26	82.53	68.62	4.31	6.30
Linoleic acid	175	0.42	15.16	7.65	2.58	33.36
Linolenic acid	175	0.02	1.40	0.24	0.14	53.54
Gadoleic acid	175	0.24	0.83	0.42	0.09	21.15
Total unsaturated fatty acids	175	56.61	88.75	77.30	4.07	5.29
Alpha-tocopherol	175	0.00	85.20	43.76	18.74	46.61
Beta + gamma-tocopherol	175	0.00	9.64	0.66	1.30	124.42
Delta-tocopherol	175	0.00	3.39	0.93	0.67	72.61
Squalene content	189	0.00	584.47	62.69	87.12	96.67
Polyphenol content	189	0.00	143.59	13.35	25.97	119.84

**Table 3:** Loadings of 21 Traits on Five Principal Components.

Trait	PC1	PC2	PC3	PC4	PC5	Communality
Seed oil content	−0.43	−0.47	0.33	0.38	0.11	0.66
Kernel oil content	−0.32	−0.60	0.37	0.34	0.17	0.74
Maximum single fruit weight	0.43	0.35	0.16	0.38	0.25	0.54
Seed yield rate of fresh fruit	−0.18	0.04	−0.18	−0.38	−0.21	0.25
Seed yield rate of dried fresh fruit	−0.40	−0.50	−0.26	0.13	0.08	0.50
Fruit length	0.60	0.33	0.20	0.30	−0.49	0.83
Fruit diameter	0.58	0.48	0.10	0.47	−0.20	0.84
Fruit shape index	0.06	−0.24	0.16	−0.24	−0.50	0.40
Pericarp thickness	0.50	0.26	0.27	0.42	−0.14	0.59
Palmitic acid	−0.61	0.73	0.05	−0.02	−0.15	0.93
Stearic acid	−0.66	0.61	0.23	0.06	0.07	0.86
Oleic acid	0.32	−0.85	0.18	0.12	−0.18	0.90
Linoleic acid	0.46	0.27	−0.45	−0.21	0.38	0.68
Linolenic acid	0.51	0.05	−0.45	−0.21	0.28	0.59
Gadoleic acid	0.58	0.02	−0.23	−0.12	−0.33	0.51
Total unsaturated fatty acids	0.66	−0.72	−0.12	−0.01	0.06	0.98
Alpha-tocopherol	0.39	0.00	0.49	−0.27	0.42	0.64
Beta + gamma-tocopherol	0.26	0.01	0.65	−0.49	−0.01	0.73
Delta-tocopherol	0.29	0.12	0.63	−0.53	0.10	0.79
Squalene content	0.34	0.18	−0.11	0.27	0.43	0.42
Polyphenol content	0.01	0.10	0.28	0.12	0.42	0.28
Variance explained (%)	20.0	17.5	10.6	9.1	7.8	
Cumulative (%)	20.0	37.5	48.1	57.2	65.0	

## Data Availability

The data are available from the corresponding author upon reasonable request.

## Supplementary Materials

The following supporting information can be downloaded at https://www.mdpi.com/article/10.3390/metabo16070512/s1. Table S1: Principal component scores and comprehensive scores of 189 *C. oleifera* individuals. Table S2: Information on 189 germplasm samples. Table S3: Method validation parameters for chemical analyses.

## Abbreviations

The following abbreviations are used in this manuscript:

PCA	Principal Component Analysis
RP-HPLC-FLD	Reversed-Phase High-Performance Liquid Chromatography with Fluorescence Detection
GC–MS	Gas Chromatography–Mass Spectrometry
BHT	Butylated Hydroxytoluene
TPC	Total Polyphenol Content
SPE	Solid-Phase Extraction
SD	Standard Deviation
CV	Coefficient of Variation
