# Supplementary material for: Comprehensive Evaluation of Fruit Traits and Altitudinal Adaptability of 189 Wild Camellia oleifera Germplasms in East Guizhou, China
Source: Metabolites. 2026 Jul 22;16(7):512. doi: 10.3390/metabo16070512 (PMC13413500; doi:10.3390/metabo16070512)
Supplement: Supplementary file 1 [file metabolites-16-00512-s001.zip › TableS1.pdf]

Table S1. Principal component scores and comprehensive scores of 189 Camellia oleifera individuals.

Sample	PC1	PC2	PC3	PC4	PC5	Comprehensive score
CL40	4.31	4.45	1.08	4.85	6.36	4.15
MJX2	5.16	1.59	-0.45	2.58	2.15	2.56
Q8	3.95	2.37	-0.15	0.90	0.18	1.98
YP599	3.28	3.19	-0.67	-0.68	0.35	1.71
ST743	3.74	2.99	0.99	-1.32	-2.31	1.66
MY101	2.63	1.03	0.70	2.30	1.01	1.65
BJ716	2.25	6.05	-2.94	-1.41	-0.63	1.57
BJ724	4.04	2.00	-0.57	-0.97	-0.19	1.53
YP21-5	1.55	3.52	-1.38	0.80	1.06	1.44
TR22-03	2.87	1.24	3.35	-2.48	-0.03	1.41
TR21-5	0.72	2.22	1.14	1.92	1.00	1.39
XL210-531	2.93	1.29	-0.53	1.43	-0.57	1.30
MJX3	-0.39	2.35	1.64	1.13	2.89	1.29
BJ726	1.84	0.62	1.60	1.53	-0.32	1.17
MJX4	-0.72	1.80	2.73	1.93	1.26	1.13
YP555	2.31	0.55	0.37	-0.20	1.74	1.10
MY36	0.28	3.38	0.30	-0.11	0.34	1.07
CL20	1.16	2.05	1.62	-0.67	-0.25	1.05
TR21-12	-1.30	2.37	3.73	2.28	-1.13	1.03
BJ719	2.34	0.73	-0.87	1.57	0.25	1.02
DZ1-537	-0.68	4.33	3.65	-1.56	-2.67	1.01
YP536	3.38	1.06	-1.45	-0.94	0.43	1.01
BJ723	5.77	-3.46	4.06	-1.73	-2.23	0.99
TR22-02	0.50	1.15	1.54	0.54	1.56	0.98
MJX6	1.75	-0.62	0.70	2.99	0.54	0.97
Q19	1.02	2.71	-1.16	1.38	-0.89	0.94
SQ760	1.49	1.34	0.39	1.75	-1.58	0.94
YP515	2.53	1.43	-0.09	-2.45	0.92	0.92
BJ735	3.04	1.02	-0.02	-1.23	-1.06	0.91
ST751	1.82	0.19	0.10	1.96	-0.52	0.84
MJX1	0.86	-0.38	0.86	2.37	1.09	0.77
BJ721	2.45	-0.30	-0.11	0.96	-0.58	0.72
CL27	-0.93	3.84	-0.25	-1.00	1.14	0.71
YP710	0.62	1.88	0.97	-1.90	0.79	0.68
YP586	-0.15	4.19	-0.79	-1.44	-0.69	0.67

Sample	PC1	PC2	PC3	PC4	PC5	Comprehensive score
BJ720	2.51	-0.68	0.28	0.39	-0.23	0.66
ST747	1.14	1.88	-0.39	0.98	-2.28	0.66
TR21-13	4.59	-3.10	0.44	-0.72	0.60	0.62
BJ740	2.10	0.46	-0.26	0.20	-1.28	0.60
MY027	0.66	-0.58	6.07	-3.81	0.87	0.60
MY2-510	6.24	-2.03	-4.83	-1.01	1.25	0.60
BJ732	0.09	2.89	-0.28	-0.68	-0.67	0.59
MY5	0.88	0.37	0.04	1.04	0.22	0.55
BJ737	0.45	1.25	0.40	1.34	-1.48	0.55
CL53	1.17	1.00	-0.61	0.62	-0.61	0.54
YP1-522	0.13	0.92	0.97	1.14	-0.52	0.54
BJ717	2.82	2.34	-5.62	-0.75	0.50	0.54
BJ728	1.92	-0.79	0.99	0.78	-0.92	0.53
YP22-2-4	-0.04	0.62	1.54	0.91	-0.10	0.52
TR22-2-1	0.90	0.41	-0.70	1.31	0.42	0.51
BJ725	2.37	-2.66	2.60	1.04	-0.63	0.50
BJ727	0.26	1.98	-1.30	0.85	-0.17	0.50
YP554	2.60	-1.02	-0.07	-1.20	1.28	0.50
YP22-2-5	-0.14	3.34	-0.83	-2.48	0.91	0.49
YP572	0.77	2.64	-2.18	-1.24	0.53	0.48
YP585	0.74	-0.08	-0.12	1.04	1.22	0.48
YP21-2	0.06	1.12	1.67	-0.40	-0.61	0.46
TR21-15	2.34	0.87	-1.40	-0.70	-1.49	0.45
MY061	1.31	0.55	1.09	-1.04	-1.19	0.44
ST745	1.01	0.17	3.15	-1.77	-1.59	0.43
YP41	0.98	0.74	-1.30	0.71	0.27	0.42
Q4-534	0.75	1.27	-0.62	-1.33	1.12	0.42
MY12	1.90	0.17	-1.12	0.68	-1.20	0.40
QB-541	1.22	0.85	0.20	-1.14	-0.69	0.39
BJ734	-1.53	3.81	0.23	-2.13	0.21	0.32
YP566	-0.96	-0.68	1.50	1.32	2.75	0.28
BJ718	-2.03	2.46	2.12	0.62	-1.55	0.28
MY096	2.19	-0.56	-2.64	2.09	-1.46	0.21
ST744	-1.31	2.55	0.10	-0.57	-0.21	0.19
YP21-3	0.60	-0.38	0.38	0.24	0.07	0.19
YP568	0.62	-0.55	0.04	-1.60	2.88	0.17
YP709	3.33	-3.42	0.86	2.53	-3.58	0.17

Sample	PC1	PC2	PC3	PC4	PC5	Comprehensive score
YP569	-1.10	1.73	-1.14	0.16	1.47	0.14
YP598	-0.28	0.04	2.15	0.05	-1.14	0.14
YP574	2.03	-1.41	1.90	-0.06	-3.63	0.11
YP579	-0.89	1.86	0.02	-2.23	1.55	0.10
BJ741	0.98	-1.51	0.02	1.38	-0.11	0.08
SQ782	-1.15	0.35	1.05	1.62	-0.55	0.07
MJX5	-0.39	0.28	-0.50	-0.49	2.11	0.06
SQ773	1.24	-1.96	0.66	0.52	0.22	0.06
BJ739	2.12	-2.89	1.23	2.55	-3.27	0.04
MY062	0.39	-1.36	1.15	-0.29	1.10	0.03
MY6-523	-1.35	0.87	1.54	-0.18	-0.13	0.03
YP22-2	-0.25	-0.69	0.73	1.30	-0.17	0.02
TR21-11	-0.68	-0.15	-0.35	1.10	1.25	-0.00
MY060	-2.80	2.07	0.53	0.43	1.15	-0.02
BJ736	0.89	-1.06	3.01	-4.23	0.69	-0.03
YP590	-2.00	2.19	1.44	-1.90	-0.09	-0.07
MJX9	-0.28	-1.47	0.04	2.42	0.33	-0.09
TR21-7	0.22	-0.84	0.11	0.44	-0.17	-0.10
MY046	0.31	-1.18	0.58	-0.36	0.51	-0.12
XL15-542	0.51	-0.43	0.74	-2.20	0.25	-0.12
YP587	-0.66	0.27	0.35	-0.72	0.49	-0.12
SQ768	-2.19	-0.46	2.13	1.47	1.05	-0.12
TR21-4	-0.20	-0.89	1.37	0.54	-0.99	-0.12
YP21-9	-1.08	-0.13	0.91	-0.35	1.09	-0.14
YP714	-0.58	0.37	0.71	-0.03	-1.45	-0.14
SQ765	0.73	-1.30	0.88	-1.39	0.22	-0.15
MY3-513	0.88	-1.39	-0.82	-0.38	1.07	-0.16
MY007	-0.63	-0.56	0.71	-0.21	0.42	-0.21
TR21-17	3.10	-3.78	-0.19	-1.33	0.47	-0.23
YP600	-1.24	0.89	-0.36	-0.11	-0.25	-0.25
YP562	-0.11	0.08	-1.13	-0.79	-0.20	-0.33
SQ761	-1.76	-0.20	0.77	-0.34	1.48	-0.34
TR21-14	-0.20	-1.29	-0.13	1.44	-0.96	-0.34
MY3-512	-0.07	-0.54	-1.08	-0.30	0.17	-0.36
SQ774	-1.76	-0.85	-0.10	2.50	0.57	-0.37
YP22-4	-1.39	-0.71	0.22	1.33	0.16	-0.38
BJ731	-0.20	-0.70	-0.24	1.16	-2.10	-0.38

Sample	PC1	PC2	PC3	PC4	PC5	Comprehensive score
YP547	0.95	-1.81	-0.15	-0.83	-0.40	-0.38
SQ783	-1.63	-1.27	1.82	0.16	1.09	-0.40
TR22-04	-1.86	-0.40	0.24	1.15	0.57	-0.41
ST746	-1.30	0.72	0.02	0.78	-2.66	-0.41
MY044	0.32	-2.37	-0.61	-0.69	2.58	-0.43
ST759	-0.92	0.19	-0.65	-0.97	-0.06	-0.48
ST758	-0.92	-1.14	-0.06	0.58	0.06	-0.51
SQ778	-0.99	-0.50	-0.96	0.10	0.48	-0.52
TR21-19	-0.11	-1.27	0.07	-1.26	0.10	-0.53
YP539	-7.42	6.15	0.71	0.37	-0.60	-0.53
MY043	-0.57	-1.51	-1.01	1.17	0.37	-0.53
MY066	0.41	-3.81	1.42	0.19	0.87	-0.54
SQ766	0.20	-1.52	-1.59	0.43	0.06	-0.54
YP594	-1.31	-0.96	-0.13	1.28	-0.28	-0.54
MY017	-0.35	-1.20	0.06	-1.46	0.68	-0.54
MY50	-0.74	-1.04	0.15	-0.36	-0.14	-0.55
YP563	-1.24	-0.60	-0.29	0.39	-0.17	-0.56
YP564	0.50	-1.09	-0.99	-1.39	-0.54	-0.56
BH-4	-0.32	-2.24	-0.52	0.80	0.91	-0.56
SQ772	-3.18	-0.29	1.02	1.21	1.25	-0.57
YP551	-1.01	-0.05	-1.18	-1.65	1.17	-0.61
YP22-2-2	-0.18	-0.68	-1.57	-1.37	0.52	-0.62
TR22-06	-0.74	-0.72	0.05	-1.60	-0.30	-0.67
YP580	-2.44	0.32	-0.04	1.39	-1.67	-0.68
QE517	-1.60	0.79	-1.35	-0.43	-1.06	-0.69
SQ785	-5.19	3.88	0.68	-0.40	-1.59	-0.69
SQ784	-2.80	0.19	0.49	-0.95	1.39	-0.70
MY37	-0.73	-1.84	-0.38	0.98	-0.80	-0.74
SQ794	-1.24	-1.50	0.69	-0.35	-0.18	-0.74
ST755	-0.23	-1.18	-1.76	0.19	-1.44	-0.82
TR21-6	-0.19	0.03	-3.19	-0.59	-1.45	-0.83
Q7	-2.05	1.25	-2.85	0.21	-0.84	-0.83
TR21-2	-0.45	-1.87	-1.28	0.02	0.14	-0.83
ST748	-1.68	0.08	-1.28	0.44	-1.69	-0.85
TR21-1	-2.03	-1.04	-0.63	1.75	-0.90	-0.87
YP576	-2.07	-0.10	0.50	-1.80	-0.37	-0.88
YP578	-0.32	-1.80	-1.06	-0.20	-0.92	-0.90

Sample	PC1	PC2	PC3	PC4	PC5	Comprehensive score
MY035	-1.93	-2.55	1.03	0.16	1.59	-0.90
YP553	0.16	-2.04	-1.32	-1.00	-0.52	-0.92
SQ777	-2.40	-1.20	-0.44	1.01	0.59	-0.92
YP591	-0.72	-0.20	-3.00	-0.17	-1.18	-0.93
YP595	-2.32	0.50	-1.01	-1.00	-0.39	-0.93
SQ789	-1.23	-0.81	-0.23	-1.64	-0.58	-0.93
SQ769	-1.26	-2.77	-0.88	1.03	1.53	-0.95
MY070	2.69	-4.95	-1.09	-0.60	-1.52	-0.95
MY25	-3.67	0.71	-0.81	1.13	-0.40	-0.96
SQ770	0.10	-2.32	0.02	-2.39	-0.65	-1.01
QY-3-504	-0.91	-1.88	-0.50	-1.72	0.80	-1.01
MY102	-2.14	0.49	-2.35	0.23	-1.24	-1.02
TR21-18	-0.41	-2.72	0.52	-2.94	1.08	-1.06
YP548	-2.72	-0.06	-0.03	-0.24	-1.65	-1.09
ST749	-1.45	-2.24	-0.55	-0.58	0.68	-1.14
SQ793	-1.07	-1.72	-1.77	-1.27	0.70	-1.17
SQ764	-2.41	-1.96	-0.54	0.19	1.18	-1.19
YP589	-2.35	1.82	-3.10	-1.27	-2.29	-1.19
MY1-507	-1.21	-2.24	-1.04	-1.17	0.89	-1.20
SQ791	-3.39	-1.05	-1.29	1.57	0.76	-1.22
SQ771	-1.64	-0.13	-3.49	-0.59	-0.46	-1.24
YP550	-1.70	-1.56	0.06	-3.14	1.08	-1.24
SQ787	-4.08	-1.06	0.10	1.45	0.34	-1.28
SQ779	-1.09	-3.75	-0.92	-0.86	1.60	-1.42
SQ788	-3.29	-2.23	0.19	-0.13	1.25	-1.45
YP22-3	-3.96	-2.26	0.87	0.62	0.53	-1.53
SQ776	-2.87	-2.25	-0.84	0.09	-0.02	-1.62
YP538	-4.13	-1.68	0.60	0.76	-2.48	-1.82
YP565	-2.44	-3.30	-2.03	0.33	-0.07	-1.93
