# Supplementary material for: Comprehensive Evaluation of Fruit Traits and Altitudinal Adaptability of 189 Wild Camellia oleifera Germplasms in East Guizhou, China
Source: Metabolites. 2026 Jul 22;16(7):512. doi: 10.3390/metabo16070512 (PMC13413500; doi:10.3390/metabo16070512)
Supplement: Supplementary file 1 [file metabolites-16-00512-s001.zip › TableS2.pdf]

Table S2. Information on 189 germplasm samples..

No.	Sample name	Germplasm source	Longitude	Latitude	Altitude (m)	Altitude group
1	QY-3-504	Yuping County	108°50'45"E	27°11'58"N	452.3	400-800
2	MY1-507	Yuping County	108°51'10"E	27°12'20"N	485.6	400-800
3	MY3-513	Yuping County	108°51'22"E	27°12'32"N	499.2	400-800
4	YP515	Yuping County	108°50'55"E	27°12'08"N	465.8	400-800
5	YP1-522	Yuping County	108°54'56"E	27°17'44"N	525.3	400-800
6	XL210-531	Yuping County	108°51'00"E	27°12'12"N	470.6	400-800
7	Q7-533	Yuping County	108°50'35"E	27°11'50"N	435.6	400-800
8	Q4-534	Yuping County	108°50'38"E	27°11'53"N	438.9	400-800
9	YP536	Yuping County	108°54'47"E	27°17'28"N	530.2	400-800
10	YP538	Yuping County	108°54'48"E	27°17'32"N	531.5	400-800
11	YP539	Yuping County	108°54'49"E	27°17'32"N	532.3	400-800
12	YP547	Yuping County	108°54'51"E	27°17'34"N	533.8	400-800
13	YP548	Yuping County	108°54'53"E	27°17'35"N	534.5	400-800
14	MY012 549	Yuping County	108°54'43"E	27°17'34"N	535.2	400-800
15	YP550	Yuping County	108°54'43"E	27°17'34"N	535.2	400-800
16	YP551	Yuping County	108°54'44"E	27°17'35"N	535.8	400-800
17	YP553	Yuping County	108°54'45"E	27°17'36"N	536.4	400-800
18	YP554	Yuping County	108°54'46"E	27°17'37"N	536.9	400-800
19	YP555	Yuping County	108°54'47"E	27°17'38"N	537.3	400-800
20	YP562	Yuping County	108°55'28"E	27°18'54"N	538.2	400-800
21	YP563	Yuping County	108°55'28"E	27°18'55"N	538.6	400-800
22	YP564	Yuping County	108°55'29"E	27°18'56"N	538.9	400-800
23	YP565	Yuping County	108°55'30"E	27°18'56"N	539.2	400-800
24	YP566	Yuping County	108°55'31"E	27°18'57"N	539.5	400-800
25	YP568	Yuping County	108°55'32"E	27°18'58"N	539.9	400-800
26	YP569	Yuping County	108°55'04"E	27°17'47"N	540.2	400-800
27	YP572	Yuping County	108°55'04"E	27°17'47"N	540.6	400-800
28	YP574	Yuping County	108°55'04"E	27°17'47"N	540.6	400-800
29	YP576	Yuping County	108°55'04"E	27°17'47"N	540.6	400-800
30	YP578	Yuping County	108°55'04"E	27°17'47"N	540.6	400-800
31	YP579	Yuping County	108°55'04"E	27°17'47"N	540.6	400-800
32	YP580	Yuping County	108°55'04"E	27°17'47"N	540.6	400-800
33	YP585	Yuping County	108°55'25"E	27°18'56"N	541.9	400-800
34	YP586	Yuping County	108°55'25"E	27°18'56"N	541.9	400-800
35	YP587	Yuping County	108°55'25"E	27°18'56"N	541.9	400-800
36	YP589	Yuping County	108°55'25"E	27°18'56"N	541.9	400-800

No.	Sample name	Germplasm source	Longitude	Latitude	Altitude (m)	Altitude group
37	YP590	Yuping County	108°55'25"E	27°18'56"N	541.9	400-800
38	YP591	Yuping County	108°55'02"E	27°17'44"N	542.3	400-800
39	YP594	Yuping County	108°55'02"E	27°17'44"N	542.3	400-800
40	YP595	Yuping County	108°55'02"E	27°17'44"N	542.3	400-800
41	YP598	Yuping County	108°55'02"E	27°17'44"N	542.3	400-800
42	YP599	Yuping County	108°55'02"E	27°17'44"N	542.3	400-800
43	YP600	Yuping County	108°55'02"E	27°17'44"N	542.3	400-800
44	YP709	Yuping County	108°55'02"E	27°17'44"N	542.3	400-800
45	YP710	Yuping County	108°55'02"E	27°17'44"N	542.3	400-800
46	YP714	Yuping County	108°55'02"E	27°17'44"N	542.3	400-800
47	BJ716	Bijiang District	109°06'03"E	27°45'31"N	547.6	400-800
48	BJ717	Bijiang District	109°06'03"E	27°45'31"N	547.6	400-800
49	BJ718	Bijiang District	109°06'03"E	27°45'31"N	543.6	400-800
50	BJ719	Bijiang District	109°06'03"E	27°45'31"N	543.6	400-800
51	BJ720	Bijiang District	109°06'03"E	27°45'31"N	551.3	400-800
52	BJ721	Bijiang District	109°06'03"E	27°45'31"N	551.3	400-800
53	BJ723	Bijiang District	109°06'02"E	27°45'32"N	549.1	400-800
54	BJ724	Bijiang District	109°06'02"E	27°45'32"N	549.1	400-800
55	BJ725	Bijiang District	109°05'46"E	27°45'15"N	562.7	400-800
56	BJ726	Bijiang District	109°05'46"E	27°45'15"N	562.7	400-800
57	BJ727	Bijiang District	109°05'46"E	27°45'15"N	562.7	400-800
58	BJ728	Bijiang District	109°05'46"E	27°45'15"N	559.1	400-800
59	BJ731	Bijiang District	109°05'51"E	27°45'21"N	589.1	400-800
60	BJ732	Bijiang District	109°05'56"E	27°44'59"N	503.2	400-800
61	BJ734	Bijiang District	109°05'59"E	27°44'58"N	496	400-800
62	BJ735	Bijiang District	109°05'59"E	27°44'58"N	495	400-800
63	BJ736	Bijiang District	109°05'59"E	27°44'58"N	486.8	400-800
64	BJ737	Bijiang District	109°05'59"E	27°44'58"N	487	400-800
65	BJ739	Bijiang District	109°06'01"E	27°44'59"N	486.7	400-800
66	BJ740	Bijiang District	109°06'01"E	27°44'59"N	492.8	400-800
67	BJ741	Bijiang District	109°06'04"E	27°44'57"N	480	400-800
68	ST755	Songtao County	109°19'50"E	28°02'19"N	762.1	400-800
69	ST757	Songtao County	109°19'51"E	28°02'19"N	763.2	400-800
70	ST758	Songtao County	109°19'55"E	28°02'18"N	769.1	400-800
71	ST759	Songtao County	109°19'55"E	28°02'17"N	761.5	400-800
72	BH-4	Bijiang District	109°10'00"E	27°27'00"N	656	400-800
73	ST793	Songtao County	109°19'55"E	28°02'17"N	765	400-800

No.	Sample name	Germplasm source	Longitude	Latitude	Altitude (m)	Altitude group
74	MY007	Yuping County	108°54'49"E	27°17'37"N	512.4	400-800
75	MY010	Yuping County	108°54'50"E	27°17'38"N	515.8	400-800
76	MY015 519	Yuping County	108°54'53"E	27°17'41"N	521.6	400-800
77	MY017	Yuping County	108°54'55"E	27°17'43"N	524.2	400-800
78	MY027	Yuping County	108°54'58"E	27°17'46"N	528.5	400-800
79	MY035	Yuping County	108°55'01"E	27°17'49"N	531.7	400-800
80	MY043	Yuping County	108°55'03"E	27°17'51"N	534.2	400-800
81	MY044	Yuping County	108°55'03"E	27°17'52"N	534.8	400-800
82	MY046	Yuping County	108°55'04"E	27°17'52"N	535.6	400-800
83	MY060	Yuping County	108°55'06"E	27°17'54"N	537.9	400-800
84	MY061	Yuping County	108°55'06"E	27°17'54"N	537.9	400-800
85	MY062	Yuping County	108°55'06"E	27°17'54"N	537.9	400-800
86	MY064 544	Yuping County	108°55'02"E	27°17'54"N	540.1	400-800
87	MY066	Yuping County	108°55'02"E	27°17'54"N	540.1	400-800
88	MY070	Yuping County	108°55'02"E	27°17'54"N	540.1	400-800
89	MY096	Yuping County	108°55'02"E	27°17'54"N	540.1	400-800
90	MY101	Yuping County	108°55'02"E	27°17'54"N	540.1	400-800
91	MY102	Yuping County	108°55'02"E	27°17'54"N	540.1	400-800
92	MY12	Yuping County	108°51'20"E	27°12'30"N	518.3	400-800
93	MY25	Yuping County	108°50'38"E	27°11'50"N	438.6	400-800
94	MY35	Yuping County	108°50'43"E	27°11'55"N	445.2	400-800
95	MY36	Yuping County	108°50'44"E	27°11'56"N	446.1	400-800
96	MY37	Yuping County	108°50'44"E	27°11'56"N	446.1	400-800
97	MY5	Yuping County	108°51'12"E	27°12'22"N	505.4	400-800
98	MY50	Yuping County	108°50'50"E	27°12'02"N	458.7	400-800
99	MY60	Yuping County	108°51'35"E	27°12'48"N	538.4	400-800
100	Q19	Yuping County	108°50'43"E	27°11'55"N	445.3	400-800
101	Q7	Yuping County	108°50'35"E	27°11'50"N	435.6	400-800
102	Q8	Yuping County	108°50'38"E	27°11'53"N	438.9	400-800
103	TR21-2	Yuping County	108°50'26"E	27°11'42"N	422.5	400-800
104	TR21-4	Yuping County	108°50'28"E	27°11'43"N	424.3	400-800
105	TR21-5	Yuping County	108°50'29"E	27°11'44"N	425.1	400-800
106	TR21-6	Yuping County	108°50'30"E	27°11'45"N	426.4	400-800
107	TR21-7	Yuping County	108°50'31"E	27°11'46"N	427.2	400-800
108	TR22-02	Yuping County	108°50'38"E	27°11'52"N	435.2	400-800
109	TR22-03	Yuping County	108°50'43"E	27°11'57"N	440.6	400-800
110	TR22-04	Yuping County	108°50'52"E	27°12'05"N	450.8	400-800

No.	Sample name	Germplasm source	Longitude	Latitude	Altitude (m)	Altitude group
111	TR22-06	Yuping County	108°50'50"E	27°12'07"N	450.8	400-800
112	TR22-2-1	Yuping County	108°50'56"E	27°12'10"N	455.2	400-800
113	YP21-1	Yuping County	108°54'46"E	27°17'28"N	405.3	400-800
114	YP21-2	Yuping County	108°54'45"E	27°17'28"N	408.6	400-800
115	YP21-3	Yuping County	108°54'45"E	27°17'28"N	411.2	400-800
116	YP21-5	Yuping County	108°54'51"E	27°17'30"N	419.6	400-800
117	YP21-6	Yuping County	108°54'52"E	27°17'30"N	419.6	400-800
118	YP21-9	Yuping County	108°54'52"E	27°17'31"N	419.6	400-800
119	YP22-2	Yuping County	108°54'48"E	27°17'38"N	432.3	400-800
120	YP22-2-2	Yuping County	108°54'29"E	27°18'54"N	428.7	400-800
121	YP22-2-4	Yuping County	108°50'41"E	27°11'54"N	442.1	400-800
122	YP22-2-5	Yuping County	108°50'41"E	27°11'54"N	442.1	400-800
123	YP22-3	Yuping County	108°50'37"E	27°11'52"N	438.9	400-800
124	YP22-4	Yuping County	108°50'34"E	27°11'49"N	435.6	400-800
125	YP41	Yuping County	108°50'46"E	27°11'58"N	450.2	400-800
126	DZ1-537	Yuping County	108°51'34"E	27°12'47"N	537.2	400-800
127	HGYC 521 708	Yuping County	108°51'24"E	27°12'34"N	521.4	400-800
128	MY2-510	Yuping County	108°51'04"E	27°12'14"N	492.3	400-800
129	MY3-512	Yuping County	108°51'09"E	27°12'20"N	498.7	400-800
130	QE517	Yuping County	108°51'19"E	27°12'29"N	517.3	400-800
131	QB-541	Yuping County	108°51'37"E	27°12'50"N	541.2	400-800
132	MY6-523	Yuping County	108°51'14"E	27°12'24"N	508.9	400-800
133	XL15-542	Yuping County	108°50'48"E	27°12'00"N	455.3	400-800
134	XGYC	Yuping County	108°50'52"E	27°12'04"N	460.5	400-800
135	CL20	Yuping County	108°50'15"E	27°11'32"N	400.6	400-800
136	CL23	Yuping County	108°50'18"E	27°11'35"N	405.3	400-800
137	CL27	Yuping County	108°50'22"E	27°11'38"N	410.8	400-800
138	CL40	Yuping County	108°50'25"E	27°11'41"N	415.2	400-800
139	CL53	Yuping County	108°50'28"E	27°11'44"N	420.6	400-800
140	ST743	Songtao County	109°18'15"E	28°15'50"N	923.3	800-1200
141	ST744	Songtao County	109°18'15"E	28°15'50"N	928.1	800-1200
142	ST745	Songtao County	109°18'56"E	28°16'02"N	938.4	800-1200
143	ST746	Songtao County	109°18'57"E	28°16'01"N	944	800-1200
144	ST747	Songtao County	109°18'57"E	28°16'01"N	944	800-1200
145	ST748	Songtao County	109°18'58"E	28°16'01"N	939	800-1200
146	ST749	Songtao County	109°18'58"E	28°16'01"N	939	800-1200
147	ST751	Songtao County	109°18'50"E	28°16'52"N	1171.8	800-1200

No.	Sample name	Germplasm source	Longitude	Latitude	Altitude (m)	Altitude group
148	SQ760	Shiqian County	107°59'28"E	27°23'27"N	904.5	800-1200
149	SQ761	Shiqian County	107°59'28"E	27°23'27"N	909	800-1200
150	SQ764	Shiqian County	107°59'29"E	27°23'27"N	910	800-1200
151	SQ765	Shiqian County	107°59'56"E	27°22'20"N	923	800-1200
152	SQ766	Shiqian County	108°39'59"E	27°53'02"N	940	800-1200
153	SQ768	Shiqian County	107°59'58"E	27°22'18"N	945	800-1200
154	SQ769	Shiqian County	107°59'59"E	27°22'17"N	958	800-1200
155	SQ770	Shiqian County	107°59'59"E	27°22'17"N	962	800-1200
156	SQ771	Shiqian County	108°00'00"E	27°22'18"N	968	800-1200
157	SQ772	Shiqian County	108°00'02"E	27°22'18"N	973	800-1200
158	SQ773	Shiqian County	108°00'02"E	27°22'18"N	984	800-1200
159	SQ774	Shiqian County	108°00'02"E	27°22'18"N	985	800-1200
160	SQ775	Shiqian County	108°00'06"E	27°22'21"N	979	800-1200
161	SQ776	Shiqian County	108°00'09"E	27°22'17"N	980	800-1200
162	SQ777	Shiqian County	108°00'09"E	27°22'17"N	980	800-1200
163	SQ778	Shiqian County	108°00'16"E	27°22'10"N	1018	800-1200
164	SQ779	Shiqian County	108°00'18"E	27°22'11"N	1028	800-1200
165	SQ782	Shiqian County	108°00'19"E	27°22'11"N	1034	800-1200
166	SQ783	Shiqian County	108°00'25"E	27°22'09"N	1092	800-1200
167	SQ784	Shiqian County	108°00'26"E	27°22'08"N	1112	800-1200
168	SQ785	Shiqian County	108°00'29"E	27°22'03"N	1157	800-1200
169	SQ787	Shiqian County	108°00'29"E	27°22'04"N	1159	800-1200
170	SQ788	Shiqian County	108°00'29"E	27°22'04"N	1162	800-1200
171	SQ789	Shiqian County	108°03'15"E	27°24'30"N	1149	800-1200
172	SQ791	Shiqian County	108°05'45"E	27°26'20"N	1147	800-1200
173	SQ794	Shiqian County	108°08'10"E	27°28'05"N	1157	800-1200
174	MJX1	Shiqian County	108°10'30"E	27°29'45"N	1145	800-1200
175	MJX2	Shiqian County	108°12'45"E	27°31'20"N	1138	800-1200
176	MJX3	Shiqian County	108°15'20"E	27°33'15"N	1085	800-1200
177	MJX4	Shiqian County	108°17'40"E	27°35'00"N	1072	800-1200
178	MJX5	Shiqian County	108°20'15"E	27°37'10"N	1025	800-1200
179	MJX6	Shiqian County	108°22'30"E	27°39'00"N	1018	800-1200
180	MJX9	Shiqian County	108°24'45"E	27°40'45"N	985	800-1200
181	TR21-1	Shiqian County	108°14'50"E	27°32'53"N	1095	800-1200
182	TR21-11	Shiqian County	108°00'22"E	27°22'09"N	1055	800-1200
183	TR21-12	Shiqian County	108°00'20"E	27°22'10"N	1038	800-1200
184	TR21-13	Shiqian County	108°23'28"E	27°39'47"N	1015	800-1200

No.	Sample name	Germplasm source	Longitude	Latitude	Altitude (m)	Altitude group
185	TR21-14	Shiqian County	108°00'12"E	27°22'14"N	992	800-1200
186	TR21-15	Shiqian County	108°00'00"E	27°22'18"N	968	800-1200
187	TR21-17	Shiqian County	107°59'58"E	27°22'18"N	945	800-1200
188	TR21-18	Shiqian County	107°59'56"E	27°22'20"N	928	800-1200
189	TR21-19	Shiqian County	107°59'29"E	27°23'27"N	912	800-1200
